# Monitoring of Diabetes Mellitus Using the Flash Glucose Monitoring System: The Owners’ Point of View

**DOI:** 10.3390/vetsci10030203

**Published:** 2023-03-07

**Authors:** Mariachiara Re, Francesca Del Baldo, Antonio Maria Tardo, Federico Fracassi

**Affiliations:** Department of Veterinary Medical Sciences, University of Bologna, Ozzano dell’Emilia, 40064 Bologna, Italy

**Keywords:** continuous glucose monitoring system, quality of life, diabetic pet owners, blood glucose curves, diabetes mellitus

## Abstract

**Simple Summary:**

The flash glucose monitoring system has recently become one of the most common monitoring methods in diabetic dogs and cats. The aim of this study was to evaluate the impact of the flash glucose monitoring system on diabetic pet owners’ quality of life and the satisfaction related to its usability. Fifty diabetic pet owners who used at least one flash glucose monitoring system on their diabetic pet were asked to answer a 30-question survey. A total of 92% of diabetic pet owners reported that their pet had better diabetes control since using the device, while the most challenging aspects were ensuring proper sensor fixation during the wearing period and preventing premature detachment and costs related to its long-term use. In conclusion, the flash glucose monitoring system is considered by diabetic pet owners to be easy to use and less stressful compared to blood glucose curves, while also enabling better glycemic control. Nevertheless, costs related to its long-term use might be difficult to sustain.

**Abstract:**

The flash glucose monitoring system (FGMS) has recently become one of the most common monitoring methods in dogs and cats with diabetes mellitus. The aim of this study was to evaluate the impact of FGMS on the quality of life of diabetic pet owners (DPOs). Fifty DPOs were asked to answer a 30-question survey. More than 80% of DPOs considered FGMS easier to use and less stressful and painful for the animal compared to blood glucose curves (BGCs). Overall, 92% of DPOs reported that their pet had better diabetes control since using FGMS. The most challenging aspects of using the FGMS were ensuring proper sensor fixation during the wearing period (47%), preventing premature detachment (40%), and purchasing the sensor (34%). Moreover, 36% of DPOs reported that the device cost was difficult to afford in the long term. Comparing dogs and cats, a significantly higher number of dogs’ owners found the FGMS to be well-tolerated (79% vs. 40%), less invasive than BGCs (79% vs. 43%), and easier to maintain in situ (76% vs. 43%). In conclusion, FGMS is considered by DPOs to be easy to use and less stressful compared to BGCs, while enabling better glycemic control. Nevertheless, the costs related to its long-term use might be difficult to sustain.

## 1. Introduction

Dogs and cats with diabetes mellitus (DM) are frequently treated with exogenous insulin and a specific diet and require regular monitoring to ensure appropriate dosing [1]. In recent years, glucose monitoring has been revolutionized by the advent of continuous glucose monitoring systems (CGMSs). According to the author’s experience, these systems are progressively replacing the use of blood glucose curves (BGCs) and are nowadays one of the most widely used monitoring methods for diabetic pets. The FreeStyle Libre^®^ flash glucose monitoring system (FGMS, Abbott Laboratories Ltd., Chicago, IL, USA) is a commonly used CGMS, thanks to its easy-to-use and long sensor lifespan. This device measures interstitial glucose (IG) concentration, which correlates well with blood glucose (BG) [2,3]. However, a lag time occurs between changes in BG and IG, and the latter also is affected by local factors specific to the tissue in which it is measured [4,5]. In dogs, the FGMS provides detailed IG profiles, allowing for the more accurate detection of nadir and hypoglycemic episodes as compared to BGCs generated by a portable blood glucose meter (PBGM) [6]. It also allows for the detailed identification of the glycemic excursions occurring throughout the day or on different days [7]. In veterinary medicine, it is generally accepted that owner compliance is essential for successfully treating DM [8]. The disease and the treatment commitments are likely to have a considerable impact on owners’ daily routines and quality of life (QoL) and might represent a significant temporal, financial, and emotional burden. In support of this, a recent study showed that more than 30% of diabetic pet owners (DPOs) euthanize their pets due to the negative impact of DM management on their lifestyle [8]. For this reason, it is crucial to consider the impact of DM management and of the different monitoring methods on the QoL of DPOs. In veterinary medicine, the impact of a particular monitoring method on the QoL of DPOs has rarely been investigated. In one study, the use of home blood glucose monitoring was associated with positive changes in the QoL parameters of cats and their owners and significant glycemic improvements [9]. In two recent studies, DPOs were asked to complete a questionnaire regarding their experience with the FGMS [10,11], while a third one has evaluated owner satisfaction with the use of an FGMS through a questionnaire containing 16 yes-or-no questions [12]. The FGMS was considered to be easy to use by DPOs and provided great satisfaction [10,11,12]. Moreover, in human medicine, the use of an FGMS positively influences the QoL of diabetic patients since it significantly reduces the risk of hypoglycemic episodes, which negatively impact the QoL of diabetic patients [13]. Despite the fact that the convenience of the use of an FGMS has been sporadically addressed in previous canine and feline studies, no studies have evaluated the impact on the QoL associated with the use of an FGMS on DPOs. Therefore, the aim of this study was to investigate the impact of an FGMS on diabetic pet owners’ QoL and the satisfaction related to its usability.

## 2. Materials and Methods

### 2.1. Participants and Questionnaire

Diabetic pet owners whose animals were admitted to the Veterinary Teaching Hospital of the University of Bologna from July 2021 to September 2022 were asked to complete an online survey (Google Form, https://forms.gle/GHT2y6J1FTzKmwaX6, accessed on 1 December 2022). Owners were considered to be eligible for inclusion in the study if they had used at least one FGMS. The survey was made up of thirty questions, including multiple-choice (M) questions (5/30), single-option questions (S) (20/30), and free-text statements (F) (5/30). The survey was divided into three categories: (1) questions related to the technical use of the FGMS (Table 1), (2) a comparison between the use of an FGMS and the generation of BGCs (Table 2), and (3) the impact of an FGMS on diabetic pets and the QoL of DPOs (Table 3).

### 2.2. FGMS

The FGMS used by the owners was FreeStyle Libre Abbott^®^. This device is available online via the manufacturer’s official website. Its technical features and the application procedures have been described in previous studies [5,14]. Scanning using the sensor needs to be carried out at least every 8 h; it automatically records the IG values every fifteen minutes. The IG trends are transferred from the sensor to a reader when the user brings the handheld reader into close proximity to the sensor. The FreeStyle Libre Link^®^ mobile app can be used as an alternative to the reader. The reader stores the data for 90 days, and, if the scans are performed using the FreeStyle Libre Link^®^ app (software version 2.8.1.6120, Abbott Laboratories Ltd., Chicago, IL, USA), the glucose values are automatically uploaded to Libreview^®^ (https://www.libreview.com, accessed on 1 December 2022) when the phone is connected to the Internet. Libreview^®^ is a free, secure, cloud-based diabetes management system provided by Abbott. The system generates summary glucose reports from the uploaded sensor data, readily available for consultation by healthcare providers. The report provides a graphical trace of the glucose values of a 24 h period, allowing access to previous glucose data.

### 2.3. Statistical Analysis

Statistical analysis was carried out using a commercially available software program (MedCalc Software Ltd., Ostend, Belgium, version 20.121). Owing to the small number of cases, the continuous variables were considered to be non-parametric, and descriptive statistics were reported as a median (minimum–maximum). The categorical variables were reported as frequencies, proportions, or percentages. The differences between dog and cat DPOs regarding the tolerability of the sensor, impact on glycemic control, stress degree related to the monitoring methods (FGMS vs. BGCs), and problems related to premature sensor detachment were compared using the Fisher’s exact test. Values of *p* < 0.05 were considered significant.

## 3. Results

### 3.1. Technical Use of the FGMS

Fifty DPOs were enrolled in the study. Of them, 29/50 (58%) were dog owners and 21/50 (42%) were cat owners. The median (range) number of FGMSs used by each DPO was 4 (1–10). The number of FGMS used by each DPO was 1 in 5 cases, 2–5 in 29 cases, 6–9 in 7 cases, and 10 or more in 9 cases. Forty-two percent of DPOs reported a premature end of the sensor within 24 h of placement due to early detachment or malfunctioning. Of them, 24/21 (76%) were dog owners and 16/21 (76%) were cat owners. Among DPOs who used only one sensor, no one reported an early detachment on the first day of use.

The use of the FGMS was proposed to the DPOs by a referral center (31/50, 62%), was recommended by the primary care veterinarian (10/50, 20%), or was discovered by the owners themselves (9/50, 18%). Forty-three percent of the DPOs understood how to use the sensor, based only on the instructions provided by the veterinarian. In contrast, 14% of them (28/50) had to find more information on the Internet regarding its use (e.g., sensor manufacturer’s website, Youtube^®^ videos, and online forums).

In 58% (29/50) of cases, the FGMS was placed exclusively by the veterinarian, while, in 42% (21/50) of the DPOs (68% of dog owners, 14/21; and 32% of cat owners, 7/21), it was placed by the owner. A total of 68% (34/50) of DPOs (70% cat owners, 24/34; and 30% dog owners, 10/34) reported that additional glue was necessary to better fix the sensor onto the skin. Of these, 26% (9/34) used a liquid medical adhesive, and 74% (25/34) used a cyanoacrylate glue. Moreover, in 88% of cases (44/50), the sensor was protected with an additional bandage (cotton and elastic bandage). The sensor lifespan reported by the manufacturer (14 days) was reached in 20% of cases.

The most widely used application area of the sensor was the dorsal aspect of the neck (78% of cases, 39/50), followed by the dorsum (18% of cases, 9/50). In one case, the sensor was applied on the shoulder blade region, and in another case, it was applied on the lumbar–sacral region. Twenty-six percent (13/50) of the DPOs changed the application area for each new sensor, by rotating between their favorite application areas. Forty-nine of the fifty DPOs (98%) used the specific FGMS mobile app as a sensor reader, while only one DPO (2%) used the handheld portable reader. The glucose values obtained using the sensor were transmitted to the veterinarian by means of the Libreview^®^ data-sharing mode in 66% of cases (33/50). The remaining DPOs shared glucose values and information regarding animal health by creating Excel files or paper notes. Twenty-five DPOs (50%) began using an FGMS within three months from the DM diagnosis, while the remaining DPOs started using it three months after (up to two years) the DM diagnosis.

### 3.2. Comparison between an FGMS and BGCs

Before using an FGMS, all the diabetic pets were monitored with BGCs carried out at home or in the hospital. In particular, all the DPOs included experienced home monitoring by performing at least one BGC at home.

**Figure 1:**
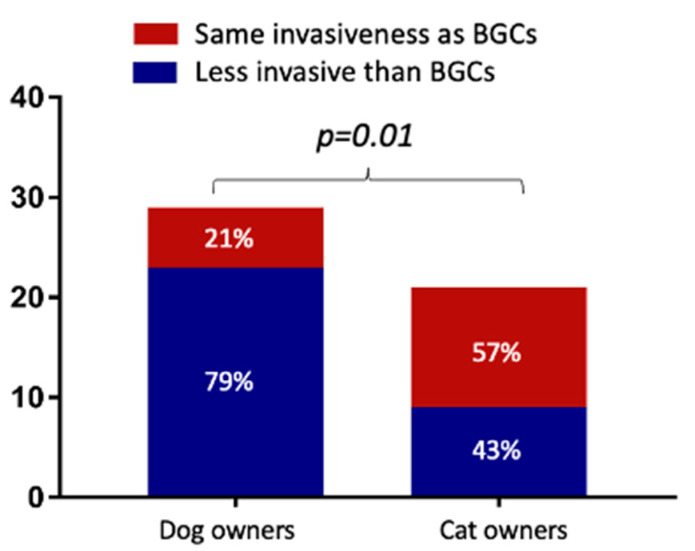
Comparison of dog and cat owners’ points of view regarding the invasiveness of the flash glucose monitoring system (FGMS) when compared to blood glucose curves (BGCs).

When comparing the use of an FGMS with a BGC, we noted that 85% (43/50) of the DPOs believed that the FGMS was easier to use than a PBGM. In addition, in 82% (41/50) of cases, the FGMS was considered less stressful and painful than a BGC. As shown by Figure 1, 79% of dog owners (23/29) considered the FGMS application to be less invasive than carrying out a BGC. In contrast, 57% of cat owners (12/21) consider it as invasive as carrying out a BGC; a significant difference was found between canine and feline DPOs (*p =* 0.01).

In the owner’s opinion, the major advantages of using the FGMS were less stress for the animal than carrying out a BGC at home or in the hospital (40/50, 79%), the possibility of obtaining more information on the glucose trend with less effort (34/50, 67%), the low invasiveness and better comfort for the animal (32/50, 64%), the ease of use (29/50, 58%), and the reliability of the results provided by the FGMS (23/50, 45%). The long-term use of the device was considered to be too expensive in 36% of cases (18/50), difficult to afford in 14% of cases (7/50), and affordable in 50% of cases (25/50)

**Figure 2:**
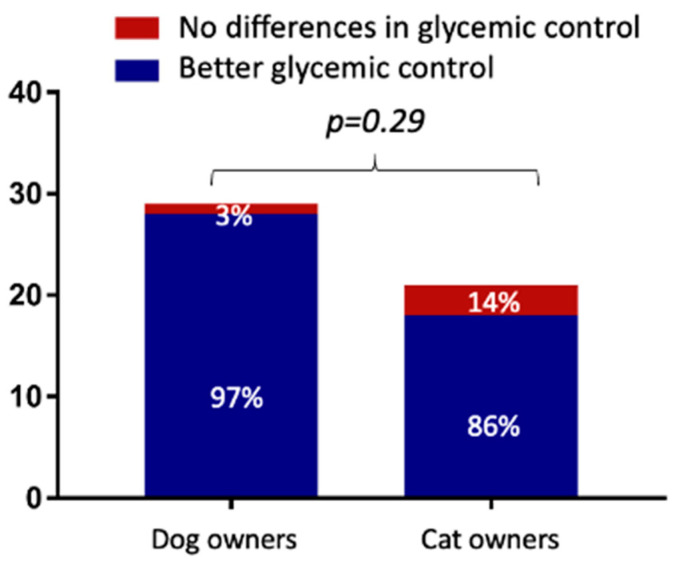
Comparison of dog and cat owners’ points of view regarding glycemic control of the flash glucose monitoring system (FGMS) when compared to blood glucose curves (BGCs).

Overall, 92% of the DPOs (46/50) believed their pet had better glycemic control since using the FGMS as a monitoring method. No differences were found between dog and cat DPOs (Figure 2; *p* = 0.29).

### 3.3. Impact of an FGMS on Diabetic Pets’ and DPOs’ Quality of Life

**Figure 3:**
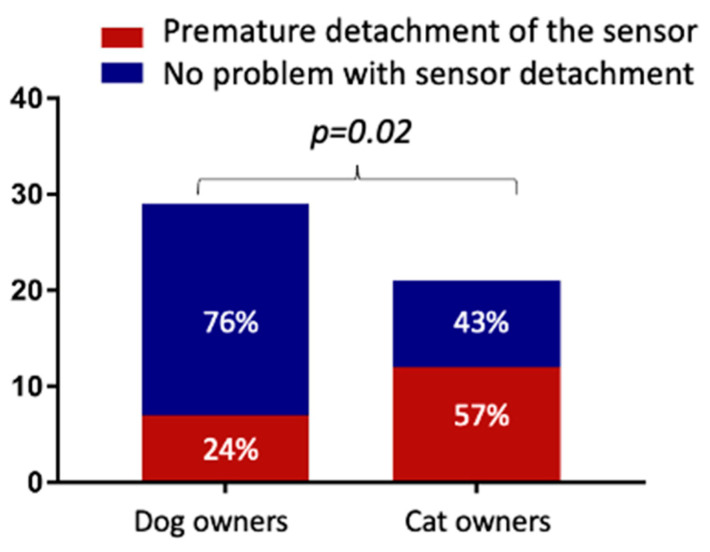
Comparison of dog and cat owners’ points of view regarding problems related to premature sensor detachment.

The most challenging and stressful aspects of using the sensor were ensuring adequate fixation during the operating period (24/50, 47%), preventing self-removal by scratching or licking (20/50, 40%), and the purchase of the sensor online (17/50, 34%). In particular, premature sensor detachment was a concern described by 57% (12/21) of cat DPOs and by 24% (7/29) of dog DPOs (Figure 3 and *p* = 0.02).

**Figure 4:**
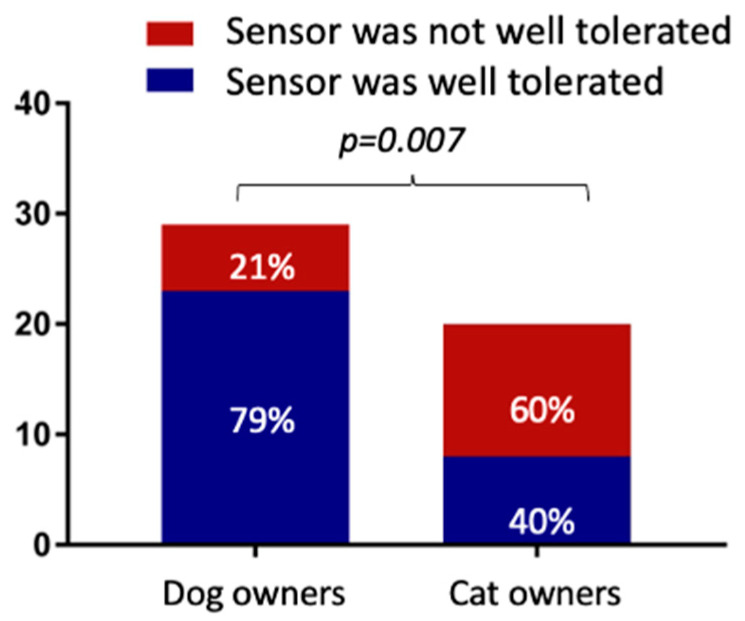
Comparison of dog and cat owners’ points of view regarding the tolerability of the sensor.

In addition, 60% (13/21) of cat DPOs reported that the sensor was not well tolerated, and a significant difference was found when compared to dog DPOs (Figure 4).

**Figure 5:**
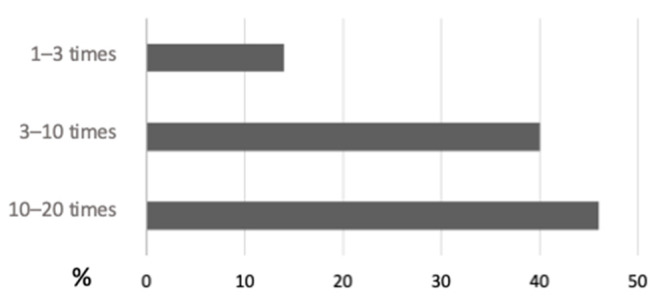
Number of sensor scans per day carried out by dog and cat owners when using the flash glucose monitoring system.

Mild-to-moderate dermatological complications after sensor removal were reported in 18% of cases (9/50). Thirty-five of the fifty DPOs (70%) stated that using an FGMS had no negative impact on their QoL. Forty-four percent of the DPOs (22/50) felt safer replacing the FGMS whenever the previous sensor stopped working. The continuous access to the glucose data generated a sense of reassurance (92%, 46/50) or increased anxiety (8%, 4/50). The number of daily scans carried out by the DPOs is shown in Figure 5. 

At the time of filling out the survey, 29/50 DPOs (58%) were still using the FGMS on their diabetic pet, and 84% of them (42/50) would continue to use it in the future. The remaining 16% of the DPOs (8/50) would not continue using the FGMS owing to its elevated cost (32/50, 64%), the difficulty of buying it (9/50, 18%), and the excessive stress for the animal (3/50, 6%). Forty-seven of the fifty (94%) DPOs would recommend the FGMS to other owners of diabetic pets.

## 4. Discussion

The FGMS is an increasingly widespread monitoring method for DM in veterinary patients. The aim of this study was to investigate the impact of an FGMS on DPOs’ QoL and the satisfaction related to its usability. According to the present results, using an FGMS as a monitoring tool provided better glycemic control than BGCs. Moreover, continuous access to the glucose data generated a sense of reassurance in the majority of the DPOs. Despite this, the main drawbacks reported by DPOs were the increased anxiety related to the possibility of having continuous access to their diabetic pet glucose values and the costs related to its use. An FGMS is designed to be worn for fourteen days. Despite this, one of the most negative aspects described by the DPOs was the reduced sensor lifespan. This was especially true in diabetic cats, and the present results are in agreement with those of previous studies [5,10,15]. In contrast, the reduced sensor lifespan was less frequently reported by dog owners. These results are in agreement with those observed in previous studies in which the maximal duration of the FGMS (14 days) was reached in about 70% of cases [12,14]. In fact, the premature detachment of the sensor represents one of the most frequent complications in diabetic cats, with a median sensor wearing time ranging from 5 to 10 days [5,7,10,16]. For this reason, in cats, to extend the sensor-wearing time, it might be advisable to additionally secure the sensor by using more glue. In the present study, approximately two-thirds of the DPOs used additional glue to extend the sensor-wearing time. This was more common among cat owners. The most used type of glue was cyanoacrylate (a multipurpose non-medical glue) due to its low cost and easy availability. Liquid medical adhesive, which is generally applied to fix dressings, patches, and some medical devices, was used in a minority of cases. Despite this, in the present study, only 20% of the sensors reached the working life of 14 days reported by the manufacturer for diabetic patients. The use of skin stitches has recently been described as a method for securing the sensor in cats [16]. In the authors’ cases, skin stitches were not used, mainly due to the excessive invasiveness of the procedure and the need to perform it exclusively in the hospital.

Almost half of the DPOs (mainly dog owners) were able to apply the sensor on their own at home. This represented an important factor in reducing costs in the management of diabetic pets.

Similar to recent studies, dermatologic complications associated with the use of FGMS were mild and self-limiting [6,10,12,17]. However, severe allergic contact dermatitis, caused by the adhesive part of the sensor, has been reported in diabetic people [18].

In the present study, the most common application site was the dorsal aspect of the neck. This is the area recommended by the authors’ veterinary hospital since it was the most commonly used location in validation studies [5,6,14]. Moreover, this area allows for an additional bandage (applied by almost 90% of the DPOs). The dorsum was the second most common application site, followed by the thoracic wall. In veterinary medicine, two studies have investigated the effect of the sensor location on the performance of another CGMS (Guardian Real-Time). In dogs, the IG measured in the chest site had the best correlation with blood glucose concentration as compared to the neck site; however, the sensor had the shortest lifespan [19]. Conversely, in cats, the dorsal neck area provided superior results in terms of accuracy when compared with the lateral chest-wall and knee fold [20]. Unfortunately, there are no data available as to whether different application sites could influence the performance of the FGMS in dogs and cats.

All the glucose values obtained during the sensor-wearing period were transmitted by DPOs to the attending veterinarian for his evaluation to aid in therapeutic decisions. The most widely used data-sharing mode was Libreview^®^, which is a cloud-based diabetes management system in which the glucose readings from the FGMS can be uploaded and shared with the healthcare professional team. This monitoring method allows monitoring the glucose trend by forming a graphical trace of glucose values over a 24 h period and having access to previous glucose data. Moreover, it provides some metrics, such as the average glucose, coefficient of variation (CV), and time of glucose within/below/above range. To date, in veterinary medicine, a single study addressed one of these parameters (CV) [12]; however, their practical application might increase in the future. In fact, the concept of glycemic variability is emerging in human medicine as an additional glycemic target [21], and a few studies have started to investigate its role in veterinary [22,23].

Several studies have described the accuracy and clinical utility of an FGMS in dogs and cats [6,7]. It has been demonstrated that an FGMS allows for more accurate identification of the glucose nadirs, post-prandial hyperglycemia, hypoglycemic episodes, and day-to-day variations in glycemic control as compared to BGCs. For this reason, the FGMS is being used more and more; therefore, it was decided to also evaluate the owners’ point of view. Approximately 80% of DPOs reported that the use of an FGMS was easier, less stressful, and less painful than carrying out BGCs. This could be explained by the fact that the application of the sensor is fast and painless. Furthermore, a majority of the DPOs were able to apply the sensor themselves. For obtaining a BGC, blood sampling is required, and when the BGC is not carried out at home, the animal requires hospitalization for at least 8–10 h. In addition, the possibility of assessing continuous glucose data remotely by using the Libreview^®^ system allows for insulin-dose adjustments, without taking the animal to the hospital. This aspect is particularly relevant for diabetic cats in which stress hyperglycemia is a common problem in the interpretation of the BGC. Nevertheless, unlike dog owners, cat owners considered the application of an FGMS to be as invasive as carrying out a BGC. This result could be explained by the lower tolerability of the sensor application and wearing by the cats. For this reason, the discomfort from wearing the sensor may be perceived by the DPOs as a sign of excessive invasiveness for the cat.

In the current study, 92% of the DPOs believed that their pet had better glycemic control since using the FGMS monitoring method. It was recently reported that, if DM is monitored using a PBGM, glucose fluctuations between blood glucose measurements might be missed, and this could result in erroneous insulin-dose recommendations [24,25]. Moreover, by monitoring glucose trends remotely, insulin-dose adjustments can be performed more frequently and probably more effectively than by carrying out BGCs. Therefore, in the authors’ opinion, these advantages may result in a better perception of glycemic control by DPOs. Nevertheless, these results might be biased by the fact that some dogs and cats were referred for sensor placement, as glucose readings were not possible or difficult to perform, and therefore DPOs asked for a different monitoring method.

Regarding the impact of an FGMS on the DPOs’ QoL, 92% of cases experienced a sense of reassurance in being able to continuously know the glucose values of their diabetic pet. Moreover, 42% of DPOs apply the sensor, continuously replacing each sensor at the end of its use with a new one. In veterinary medicine, an FGMS is used as an alternative monitoring method to BGCs. Therefore, in the authors’ clinical practice, they apply the sensor continuously until an optimal insulin dose is identified. Despite the fact that the majority of the DPOs felt a sense of reassurance, 8% of them reported that the chance to have continuous access to their diabetic pet’s glucose values caused increased anxiety. This was highlighted by the fact that 46% of the DPOs carried out between 10 and 20 glucose readings per day, although this is not necessary for the correct functioning of the sensor. In the authors’ opinion, anxiety could probably increase when DPOs detect low glucose values. However, this aspect was not evaluated in the present study.

The other major drawbacks associated with the use of the FGMS were its cost and its availability. Currently, in the authors’ country, the FGMS can only be purchased online via the official website of the manufacturer. This aspect is particularly challenging for the elderly or for those who are not familiar with the use of the Internet. In fact, 34% of DPOs stated that availability was one of the most negative aspects associated with the use of the device. Based on these results, the possibility of buying the sensor not only online but also through other sellers could probably make it more usable by all types of DPOs.

In addition to this, in 37% of the cases, the long-term use of the device was considered too expensive.

This was in agreement with previous studies in which, despite the elevated degree of satisfaction, the cost was reported to be a main drawback [10,12]. Therefore, this seems to be a common problem in different countries. Nevertheless, despite the disadvantages reported, 70% of the DPOs reported that using an FGMS had no negative impact on their QoL; this was in agreement with previous studies in human medicine in which the continuous use of an FGMS was associated with an improved QoL in diabetic patients [26,27,28,29]. Moreover, Overend et al. reported that an FGMS had a positive impact on psychological well-being and self-esteem since patients with type 1 DM experienced more control over their BG values [30].

In total, 84% of the DPOs stated that they would continue to use the device in the future, and 94% of them would recommend it to other DPOs. These data suggest that the overall good DPO satisfaction and owner perceptions of the advantages of FGMS outweigh the disadvantages.

The present study had some limitations, including the small sample size, its retrospective nature, and the fact that the survey used was not previously validated. Another limitation of this study is that the degree of stress of the diabetic pet and the DPOs’ QoL were evaluated subjectively and not through specific scores. However, the main limitation was that all the diabetic patients included were monitored at a referral center. In fact, thanks to the specialist medical staff, the DPOs were well-instructed regarding the use of the sensor and how to interpret the glucose data. This might have positively influenced the present results. For this reason, additional studies, also including diabetic pets managed by primary care veterinarians, are needed.

## 5. Conclusions

In conclusion, the FGMS was considered easy to use by the DPOs and less stressful when compared to BGCs, while enabling better glycemic control. Moreover, the possibility of having continuous access to the glucose data generated a sense of control in the DPOs. Nevertheless, the cost related to its long-term use might be difficult to sustain. Additional reported drawbacks were the availability of the sensor and the increased sense of anxiety of the DPOs. Finally, in cats, premature detachment and poor tolerability of the device are frequent concerns.

## Figures and Tables

**Table 1:** Questions related to the technical use of the flash glucose monitoring system (FGMS).

Question	Answer Form
When did you start using the FGMS monitoring method?	F
How many devices has your pet used so far?	F
How many devices could not be used due to operating problems or early detachment (within 24 h of insertion)?	F
Who proposed the FGMS to you?	S
Do you think that the information provided has been useful to understand and use the device?	S
Who applies the sensor?	S
How is the FGMS fixed to the skin?	S
Once applied, is the sensor protected by a bandage?	S
In which body area is the FGMS usually applied?	M
Is the application area of the sensor always the same?	S
How are the glucose data of your animal transmitted to the vet?	S
How long after your pet’s diagnosis of diabetes did you start using the FGMS?	F

**Table 2:** Questions related to the comparison between the use of flash glucose monitoring system (FGMS) and the generation of blood glucose curves (BGCs).

Question	Answer Form
What are the main advantages related to the use of the sensor?	M
Can you explain how your pet’s blood glucose was monitored [if you haven’t used the FGMS since the onset of the disease]?	M
Compared to BGCs, do you think that FGMS is an easier monitoring method?	S
Compared to BGCs, do you think that FGMS provides better glycemic control?	S
Compared to BGCs, do you think that the FGMS is less stressful?	S

**Table 3:** Questions related to the impact of a flash glucose monitoring system (FGMS) on the quality of life (QoL) of diabetic pets and diabetic pet owners (DPOs).

Question	Answer Form
What are the main drawbacks of using the sensor?	M
Do you think an FGMS allows better glycemic control?	S
Do you think an FGMS is well tolerated by your pet?	S
Does an FGMS have a negative impact on your QoL?	S
How many times a day do you scan the sensor?	S
How often do you apply the FGMS to your diabetic pet?	S
How do you feel about continuously accessing your pet glucose values?	S
What do you think about costs related to the use of FGMS?	S
Are you currently using an FGMS on your diabetic pet?	S
Will you use an FGMS again?	S
Why? [If you gave a negative answer to the previous question]	M
Would you recommend the FGMS to other DPOs?	S
Express you opinion	F

## Data Availability

The data presented in this study are available in the manuscript.
